# Acute Laryngitis

**Published:** 1883-09-20

**Authors:** C. E. Nelson

**Affiliations:** N. Y.


### ACUTE LARYNGITIS.

By Dr. C. E. Nelson, N. Y.

Common inflammation of the windpipe is not frequently met with. A case of great severity occurred in the practice of Dr. Schultze in the summer of last year, in this city. The patient, a stout man, in a few days got seriously worse, when on July 15th (a fearfully hot day), the day on which I was called in consultation, he seemed to be totally unconscious of all his surroundings, as far as external signs and appearances were eoncerned. For about three days he had not answered when spoken to; eyes tightly closed; and, seemingly, did not change his position in bed day in and day out. I saw him with Dr. S. at 3 p. m.; he seemed to be in such a serious condition that, upon consultation, we deemed it advisable not to trouble him much with medicine, but concluded simply to order brandy and ammonia. The next visit together, at 9 P. M., the family seemed to consider he was easier; calomel with opium (and no local treatment whatever) were then prescribed.

July 16th.—Morning visit. They said he passed the night pretty fairly. With the larynx swollen and inflamed, there was no getting any information out of the patient himself. Before our visit this morning he had had two slight convulsive tremors, showing his dangerous condition. Very soporific; eyes tightly closed all the time: patient lying on his back. With great difficulty we got him to open the eyes once, when the pupils were seen to be dilated; however, the lids were closed firmly again, remaining so. Temperature and pulse normal; feet and legs cold. The heat of the room was so stifling that the window had to be opened a little behind the shade. Pulse weaker in stroke; tongue moist, but brown. He breathes normally. On auscultation, respiration was found to be normal.

*Treatment.*—Six leeches. Calomel with opium [this sounds very much like the old-fashioned anti-phlogistic times; but it does not matter how you get well, as long as you do get well]; also, cherry brandy. Giving brandy in such a case as this would have been considered a heresy forty years since; but stimulation, undoubtedly, is often a useful adjuvant.

9 P. M.—Evening visit. The people say he is better. Has had a dark passage from the bowels. A dose of Dover's powder for the night. [Dover's powder!! Let us go back to the time of the Crusades at once.] However, Crusades or not, next morning the patient recognized his medical attendants, talked in a pleasant manner, and the brunt of the battle between life and death was over. A nice pulse; nice moist skin; tongue moist. On the next day afterwards, on the 18th, a simple mixture was prescribed, such as is given for coughs. This practically ended the case.

It is very doubtful if the prevailing quinine and iron treatment would have saved this man. Fashion is a very good thing in ladies' bonnets; but we doubt if it be a safe guide for our bodily ailments.—*The Planet.*

